# Investigating the Morphology of the Nasal Cavity for Nasal Reconstruction Using Cone Beam Computed Tomography Images: Morphology of the Nasal Cavity for Nasal Reconstruction

**DOI:** 10.31661/gmj.v13iSP1.3522

**Published:** 2024-12-31

**Authors:** Aisan Ghaznavi, Sina Ilkhani, Asma Sodaii, Mohammad Jafari Heydarlou

**Affiliations:** ^1^ Department of Oral and Maxillofacial Radiology, School of Dentistry, Urmia University of Medical Sciences, Urmia, Iran; ^2^ Department of Oral and Maxillofacial Surgery, School of Dentistry, Urmia University of Medical Sciences, Urmia, Iran

**Keywords:** Nasal Morphology, Cone Beam Computed Tomography, Nasal Reconstruction, Nasal Cavity, Facial Index

## Abstract

**Background:**

Facial reconstruction is the procedure of rebuilding a face onto an anonymous skull to aid identification in forensic and archaeological cases. This study investigated the morphology of the nasal cavity for reconstruction by using cone beam computed tomography images.

**Materials and Methods:**

In this retrospective cross-sectional study, pre-treatment CBCT images of 220 adults were selected by random sampling from the records of orthodontic clinical data between January 2022 and November 2023. The three-dimensional parameters of the nasal soft structures and hard structures were measured.

**Results:**

Of 220 CBCT images, 198 cases (61.1% females and 38.9%males) were examined in the final analysis after meeting inclusion criteria. TStatistically significant sex differences were observed in nasal length (males: 50.79±4.78 mm, females: 45.28±4.18 mm; P0.05), nasal depth (males: 23.54±2.43 mm, females: 26.05 ± 3.53 mm; p 0.05), and nasal height (males: 49.81±4.30 mm, females: 55.02±4.49 mm; P0.05). The nasolabial angle was significantly higher in males (98.21°±8.34°) compared to females (89.71°±7.37°; P0.05). Conversely, the nasal tip angle was significantly higher in females (77.18°±8.45°) than in males (71.54°±8.20°; P0.05). A statistically significant difference was also observed in the nasal upward tip angle between males (23.8 ±3.10°) and females (20.45°±2.98°; P0.05).

**Conclusion:**

This study revealed significant sex-based variations in nasal parameters. Males exhibited greater nasal length, depth, and nasal tip angle compared to females.

## Introduction

The nasal cavity, a intricate air-filled chamber, exerts a critical influence on three key functions: respiration, olfaction (smell), and facial aesthetics and does a vital position within the facial skeleton [1]. Although relatively stable during adolescence [2], nasal development concludes by age 16 in females and 18 in males [3]. The intricate shape of the nasal cavity, defined by various bone and cartilage structures, plays a role in airflow, drainage, and ultimately, how well we breathe [4]. Trauma, birth defects, and tumors can disrupt this form, causing problems with both function and appearance [5].

Nasal reconstruction surgery exists to restore normalcy in these situations, aiming to improve both breathing and facial aesthetics [6].

Facial reconstruction has been extensively employed for craniofacial recognition and identification, as evidenced by numerous investigations [7]. A substantial body of research has quantified the relationship between the skull’s skeletal structure and the overlying facial soft tissues [8][9][10]. This methodology, adaptable to the available skeletal remains and their condition, can be a valuable tool in aiding case identification, particularly in large-scale disasters. Prior studies have primarily relied on cadaveric analyses to investigate the intricate link between facial hard and soft tissues [11][12]. Notably, nasal morphology exhibits high variability and plays a significant role in facial recognition due to its unique characteristics [13].

Therefore, accurate prediction of the nasal tip has the potential to enhance the overall accuracy of facial reconstructions for identification purposes [14]. The prediction of nasal morphology from the skull has been a topic of considerable investigation, as evidenced by numerous reports [15][16]. Evaluation of nasal morphology has historically relied on various techniques, including morphometry, photogrammetry, radiography, and more recently, three-dimensional (3D) imaging modalities [17][18].

Traditional methods such as lateral cephalometry and plain radiography provided limited two-dimensional (2D) views, hindering the accurate assessment of the inherently complex 3D structures of the nasal cavity. However, the emergence of cone beam computed tomography (CBCT) has revolutionized nasal cavity imaging by offering high-resolution, 3D reconstructions. This advancement allows for a more comprehensive and accurate evaluation of nasal morphology.

To our knowledge, few studies have focused on the 3D spatial relationships between the hard tissue and overlying soft tissue of the CBCT images for nose reconstruction in Iranian ethnicity. The present study aimed to investigate the morphology of the nasal cavity for reconstruction by using cone beam computed tomography images.

## Materials and Methods

**Figure-1:**
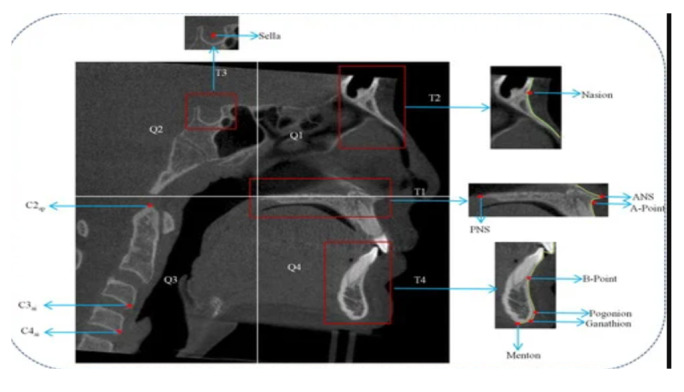
Figure 1. Reference anthropological hard tissue landmarks.

**Figure-2:**
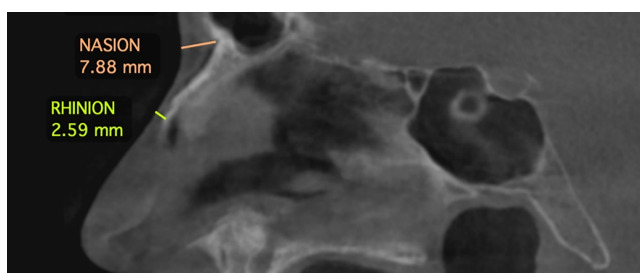
Figure 2. Reference anthropological hard tissue landmarks.

**Figure-3:**
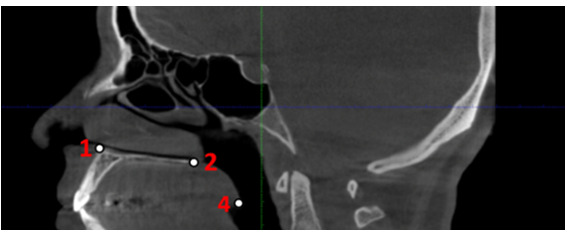
Figure 3. Reference anthropological hard tissue landmarks.

In this analytical cross-sectional study, by random sampling, pre-treatment CBCT images of 220 Iranian adults with ages more than 18 years were selected from the records of a radiography centers affiliated to Uremia University of Medical Sciences from January 2022 to November 2023.

Adherence to ethical and legal requirements was paramount, and informed consent was obtained from all participants. Ethical approval was given by the medical ethics committee of Urmia University of Medical Sciences, the following reference number was IR.UMSU.REC.1402.216.

To ensure the internal validity of the study, a strict exclusion criterion was employed. Patients with pre-existing medical conditions potentially affecting nasal morphology were excluded, including those with bone diseases, a history of nasal trauma, congenital nasal anomalies, prior nasal and facial sinus surgeries, and relevant nasal or maxillary sinus pathologies. Additionally, patients with known syndromes were excluded.

This study employed vertical cephalic radiographs with subsequent 3D reconstructions for data collection. The axial sections ensured complete visualization of the maxilla and its pterygoid processes. Scanning parameters were adjusted to optimize image quality based on individual patient characteristics. Two independent evaluators, a radiologist and a student, assessed the images using Ramixis 5.1.1 software on a computer equipped with a high-resolution (1920 x 1080 pixels) 42-inch LG monitor. Evaluations were conducted in a controlled environment free from light pollution. To assess nasal morphology, a set of established anthropological bony landmarks was utilized [15].

1- Sella (S): The midpoint of the pituitary fossa (Figure-1)

2- Nasion (N): The most anteriorly projecting point on the nasofrontal suture in the sagittal plane (Figure-1)

3- Pogonion (Pog): The most prominent anterior aspect of the mandibular symphysis (Figure-1)

4- Menton (M): The inferiormost point of the mandibular symphysis (Figure-1)

5- Rhinion (Rh): The most inferior point where the right and left nasal bones meet, essentially marking the junction between the bony and cartilaginous portions of the nasal structure in the midline (Figure-2).

6- Nasal Cavity (NC): The most laterally positioned point on the bony outline of the nasal cavity (Figure-3)

7- Anterior Nasal Spine (ANS): The anteriormost tip of the bony projection arising from the maxilla, located at the inferior border of the anterior nasal opening (Figure-3)

To comprehensively assess nasal morphology, this study additionally employed a set of established anthropological soft tissue landmarks [19] (Figure-4). These landmarks included:

1- Soft Tissue Nasion (N′): The deepest indentation in the midline between the brow ridge (glabella) and the tip of the soft tissue nose (pronasale).

2- Pronasale (Prn): The most forward-projecting point of the soft tissue nose, identified as the intersection of a perpendicular line drawn from the Frankfort Horizontal Plane (FHP) and the nasal tip.

3- Subnasale (Sn): The deepest midline point where the columella (the central pillar between the nostrils) meets the upper lip.

4- Posterior Columella Point (PCm): The rearmost point on the lower border of the nose where it curves downward to meet the philtral ridge of the upper lip.

5- Columella Point (Cm): The most anterior aspect of the columella.

6- Labrale Superius (Ls): The point marking the boundary between the skin and the mucous membrane on the upper lip.

7- Ala (Al): The outermost point on the outline of each nostril (ala).

This study employed a set of established reference planes and anthropometric variables to quantify nasal morphology [20]. These included:

1- Frankfort Horizontal Plane (FHP): A reference plane constructed by connecting the highest point on the opening of each ear canal (external auditory meatus) with the lowest point on the inferior margin of the left eye socket (orbit).

2- Nasal Length: The linear distance measured between the soft tissue nasion (N′) and the pronasale (Prn).

3- Nasal Depth: The perpendicular distance between the pronasale (Prn) and a line drawn from the soft tissue nasion (N′) to the subnasale (Sn).

4- Nasal Width (Ala-Ala): The maximum horizontal distance between the left and right alar points (outermost points of the nostrils).

5- Nasal Height (N′ - Sn): The linear distance measured between the soft tissue nasion (N′) and the subnasale (Sn).

6- Nasal Index (Ala-Ala/N-Sn): A dimensionless ratio calculated by multiplying the ratio of the maximum nasal width (ala-ala) to the maximum nasal height (N′ - Sn) by 100. This index helps categorize nasal breadth relative to height.

7- Nasolabial Angle (NLA): The angle formed at the intersection of a tangent line drawn along the posterior columella point (PCm) and a line connecting the PCm to the labrale superius (Ls) (the border between the upper lip’s skin and mucous membrane).

8- Nasal Upward Tip Angle (UNLA): The angle formed when the tangent line drawn along the posterior columella point (PCm) is extended anteriorly to intersect the Frankfort Horizontal Plane (FHP). This angle reflects the upward rotation of the nasal tip.

9- Nasal Tip Angle (NTP): The angle formed by the intersection of the axis of the nasal dorsum (bridge) and the tangent line drawn along the posterior columella point (PCm). This angle reflects the projection of the nasal tip.

### Sample Size Calculation

The sample size in the present study was calculated based on the study by Gruszka et al. [21], in which the ratio (p) of the most common changes related to the anatomical variation related to nasal cells was 54.8 and the confidence interval coefficient 95% z_(1-α/2)=1.96≈2 and the accuracy (d) for estimating the point which is the minimum important difference from the clinical aspect for the difference between the groups was estimated as 198 according to the following formula.

### Statistical Analysis

Quantitative findings were reported in the form of mean and standard deviation, and qualitative findings, which were the gender distribution of the studied subjects, were reported in the form of percentages. An independent samples t-test was used to compare the estimates between the males and females. All statistical analyses were performed using SPSS 22.0. P<0.05 was considered to be statistically significant.

## Results

**Table:** Table1. Descriptive Data and Gender Variation of Nasal Parameters

	**Male (n = 77 (38.9%))**		**Female (n = 121 (61.1%))**		**P-value**
**Mean**	**SD**	**Mean**	**SD**	<0.000
Nasal length (mm)	50.79	4.78	45.28	4.18
Nasal depth (mm)	23.54	2.43	26.05	3.53	<0.000
Nasal width (mm)	25.95	1.8	24.16	2.13	0.432
Nasal height (mm)	49.81	4.3	55.02	4.49	<0.000
Nasal Index (%)	65.67	7.61	64.88	7.15	0.467
Nasolabial angle (NLA)	98.21	8.34	89.71	7.37	0.051
Nasal upward tip angle (UNLA)	23.8	3.1	20.45	2.98	<0.000
Nasal tip angle (NTP)	78.18	8.45	71.54	8.2	<0.000

To assess intra-observer variability, a random selection of 20 CBCT images were traced twice by the researcher at a two-week interval. The Kappa coefficient was calculated, demonstrating acceptable agreement (values not shown).

Prior to commencing the main study, a pilot evaluation was conducted with one radiologist and one student examiner following their training.

The intraclass correlation coefficient was used to assess inter-observer agreement, yielding values of 0.85, 0.92, and 0.91 for nasal length, depth, and height measurements, respectively. These values indicate good to excellent reliability.

A total of 220 CBCT images were initially examined. Following application of inclusion and exclusion criteria, 198 cases were included in the final analysis. The sample comprised 121 females (61.1%) and 77 males (38.9%).

Statistically significant sex differences were observed in nasal length (males: 50.79 ± 4.78 mm, females: 45.28 ± 4.18 mm; p < 0.05), nasal depth (males: 23.54 ± 2.43 mm, females: 26.05 ± 3.53 mm; p < 0.05), and nasal height (males: 49.81 ± 4.30 mm, females: 55.02 ± 4.49 mm; p < 0.05). However, no significant sex differences were found in nasal width (males: 25.95 ± 1.80 mm, females: 24.16 ± 2.13 mm; p = 0.432) or nasal index (males: 65.67 ± 7.61%, females: 64.88 ± 7.15%; p = 0.467). The nasolabial angle was significantly higher in males (98.21° ± 8.34°) compared to females (89.71° ± 7.37°; p < 0.05). Conversely, the nasal tip angle was significantly higher in females (77.18° ± 8.45°) than in males (71.54° ± 8.20°; p < 0.05). A statistically significant difference was also observed in the nasal upward tip angle between males (23.8° ± 3.10°) and females (20.45°± 2.98°; P<0.05, Table-1).

## Discussion

Facial reconstruction is a multifaceted discipline that integrates scientific methodologies with artistic expertise to recreate soft tissues on a skull [22]. This technique offers valuable applications in forensic medicine, enabling the generation of facial approximations for identification and diagnostic purposes [23]. Furthermore, it plays a crucial role in facial reconstruction and corrective surgeries [24]. In light of this, the present study employed CBCT images to investigate nasal cavity morphology in the context of facial reconstruction.

Previous research has employed diverse methodologies and anatomical landmarks to evaluate nasal morphology and its relationship with surrounding hard and soft tissues, primarily using lateral cephalometric radiographs [17][25][15]. For instance, Aljabaa et al. investigated the influence of sex and ethnicity on nasal form and its association with other cranial structures, highlighting the importance of these factors when establishing normative values [17]. Additionally, researchers have adopted quantitative approaches to assess nasal morphology and its connections to other facial elements within distinct ethnic populations [15][25].

However, critiques have emerged regarding the application of specific methods. Lapointe et al. argued for the use of Stephan’s craniofacial norm instead of Gerasimov’s method, suggesting potential inaccuracies in the latter [26]. Furthermore, studies focusing on specific Asian populations have contributed valuable data. Other related study provided detailed information on craniofacial soft tissue thickness and nasal profile characteristics within the Chinese Xi’an Han population [27].

Advancements in dental technology have introduced innovative tools like CBCT. The increasing demand for three-dimensional (3D) imaging has propelled CBCT to the forefront of diagnostic techniques in dentistry [28]. This widespread adoption is attributed to several factors, including CBCT’s ability to generate high-resolution volumetric images of the jawbone at a cost-effective radiation dose [29]. Additionally, its compact size, affordability, and in-office or close-by availability enhance its clinical utility [30]. Nasal morphology exhibits significant variations across ethnicities [2]. Previous research has explored methods for predicting nasal projection and the position of the pronasale (the most anterior point of the soft tissue nose). These studies have established a link between nasal morphology and the relative positioning of the pronasale, subnasale (the deepest point where the columella meets the upper lip), and nasal alae (outermost points of the nostrils) on the underlying nasal cavity [2][31].

Limited research has explored the potential of CBCT images to predict nasal position through analysis of the 3D spatial relationships between the underlying skeletal structures and the overlying soft tissues [32]. This study utilized CBCT images to perform measurements of both the soft tissue nose and the corresponding hard tissue nasal aperture. The results demonstrated significant sex-based differences in nasal length, depth, and tip angle. These findings align with previous observations reported by Wang et al. [13], Prasad et al. [33], and Zamani et al. [34].

Previous research conducted on Indonesian and Scottish populations suggests that the nasal index, a ratio of nasal width to height, serves as a reliable anthropometric parameter for geographic origin estimation [29][35]. Additionally, the nasal index has established utility in forensic science due to its demonstration of sexual dimorphism, aiding in human identification [36]. While our study incorporated nasal index calculations, the results did not reveal statistically significant sex-based differences in this sample. It is important to acknowledge that craniometric parameters, including the nasal index, have been explored in prior research for sex estimation within forensic contexts [15]. However, the validity of these parameters hinges on the understanding that they can vary considerably across populations [37].

Extensive literature highlights significant variations in orbital and nasal morphology influenced by age, sex, and ethnicity [38]. These observations resonate with the findings of Vidya et al. [39] who examined South Indian skulls and Nasir et al. [40] who investigated nasal indexes across various Indian states. Their studies reported a higher nasal index in males compared to females, which is not necessarily reflected in our specific sample.

The nasolabial angle remains a valuable clinical and cephalometric parameter, playing a crucial role in assessing the soft tissue profile [41]. Research suggests population-based variations in NLA values, with Asian populations generally exhibiting higher values indicative of flatter facial profiles and more obtuse angles compared to Caucasian or African populations [42].

Our study’s findings align with this trend, demonstrating significant sex-based differences in the nasolabial angle. These observations are consistent with Kandhasamy et al., who reported higher NLA values in males compared to females [43]. The underlying cause for this sexual dimorphism is likely attributable to variations in the location of the nasolabial angle’s vertex point. Kandhasamy et al. defined the vertex as the midpoint of tangents drawn along both the columella and the upper lip. This approach positioned their vertex considerably posterior to the posterior columella point, potentially influencing the measured NLA value.

### Limitations of the Study

It is important to acknowledge that the current study focused on an Iranian population sample. Nasal morphology exhibits significant variations across ethnicities, limiting the generalizability of these findings to other populations [44].

Despite this limitation, the study offers valuable data on the relationships between the skull and facial soft tissues in the context of nasal reconstruction, along with providing prediction guidelines for this process. These findings hold relevance for applications in forensic science and anthropology. Looking forward, future research should expand its scope to investigate nasal morphology in other major ethnicities, potentially including populations within Iran. Additionally, employing larger sample sizes would be beneficial in establishing standardized norms for nasal characteristics. These efforts can ultimately contribute to improved accuracy in facial reconstruction techniques and enhanced success rates in forensic identification.

## Conclusion

This study revealed significant sex-based variations in nasal parameters. Males exhibited greater nasal length and depth compared to females. Additionally, a notable difference was observed in the nasal tip angle between the sexes, with males having a more projected nasal tip angle. The clinical significance of this study is that hard and soft tissue relation data from CBCT can be useful for predicting the position of the nose. The values of the nasal form are useful for facial reconstruction and rhinoplasty.

## Conflict of Interest

The authors have no competing interests to declare that are relevant to the content of this article.
